# New insights into the interplay between codon bias determinants in plants

**DOI:** 10.1093/dnares/dsv027

**Published:** 2015-11-05

**Authors:** S. Camiolo, S. Melito, A. Porceddu

**Affiliations:** Dipartimento di Agraria, SACEG, Università degli Studi di Sassari, Sassari, Italy

**Keywords:** codon bias, mutational bias, translational selection, plant genetics

## Abstract

Codon bias is the non-random use of synonymous codons, a phenomenon that has been observed in species as diverse as bacteria, plants and mammals. The preferential use of particular synonymous codons may reflect neutral mechanisms (e.g. mutational bias, G|C-biased gene conversion, genetic drift) and/or selection for mRNA stability, translational efficiency and accuracy. The extent to which these different factors influence codon usage is unknown, so we dissected the contribution of mutational bias and selection towards codon bias in genes from 15 eudicots, 4 monocots and 2 mosses. We analysed the frequency of mononucleotides, dinucleotides and trinucleotides and investigated whether the compositional genomic background could account for the observed codon usage profiles. Neutral forces such as mutational pressure and G|C-biased gene conversion appeared to underlie most of the observed codon bias, although there was also evidence for the selection of optimal translational efficiency and mRNA folding. Our data confirmed the compositional differences between monocots and dicots, with the former featuring in general a lower background compositional bias but a higher overall codon bias.

## 1. Introduction

The genetic code is redundant, with most amino acids encoded by two or more synonymous codons.^1–4^ The non-random use of synonymous codons is known as codon bias, and it may reflect several underlying factors, including mutational bias in the genome and translational selection. The possibility that mutational bias affects codon usage has led to the neutralist model, in which codon identity is mainly determined by nucleotide substitution patterns in the genome. In contrast, the possibility of translational selection has led to the selective model, in which the choice of synonymous codons reflects tRNA abundance^5^ to optimize the efficiency^4^ and accuracy^6^ of translation. These models are not mutually exclusive, i.e. the choice among synonymous codons may reflect a balance between selective and mutational pressures.^7^

Species-dependent differences in codon usage are well known,^8^ but recent studies have identified variations within species that must also be addressed by the neutralist and selective models. For example, the abundance of tRNA may vary during development and in response to external stimuli,^9^ suggesting that codon bias may represent an adaptive response to tRNA levels that differ among plant tissues.^10^ Nucleotide substitution patterns are also unequally distributed in the genome,^11^ e.g. there are large homogeneous blocks G + C rich sequences known as isochores in the genomes of warm-blooded vertebrates.^12,13^ Likewise, differences in nucleotide substitution patterns have been observed in rice (*Oryza sativa*), with genes expressed in the roots being predominantly G + C rich and genes expressed in seeds and leaves being predominantly A + T rich.^14^

The compositional context can also influence synonymous codon selection, a phenomenon known as context-dependent codon bias (CCDB). In mammals, bacteria and plants, the first nucleotide after each codon drives synonymous codon choice, because several dinucleotide sequences such as CG, GA and TA are under-represented.^15–17^ In plant genomes, there is also a general bias in the use of specific dinucleotides and trinucleotides in different genomic regions.^11,18^

The composition of coding sequences is determined by a complex series of interacting factors, so it is difficult to identify the relative impact of different components. A model to determine the influence of nucleotide substitution patterns on codon bias has been developed by building a new set of sequences in which the third codon position in the coding sequence is replaced with a random nucleotide from the neighbouring intergenic region.^19^ Such intergenic corrected coding sequences (ICCSs) retain the same amino acid sequence while mirroring the background nucleotide substitution pattern of the genome. Comparing the codon bias between the original coding sequence (CS) and ICCS datasets can therefore highlight the influence of the background nucleotide substitution pattern on the coding sequence composition.^19^

Although the selective model has been studied in several plant species, the impact of background composition on gene structure has been largely overlooked. Codon bias in *Arabidopsis thaliana* (Arabidopsis) tends to be associated with the composition of the 3′ flanking region in both strongly and weakly expressed genes,^20^ although the impact of selection on both classes of genes has also been recognized. Here we used the Hershberg and Petrov approach^19^ to determine the effect of background composition on the codon bias of 21 plant species while also accounting for bias in the frequencies of dinucleotides and trinucleotides. We discuss in detail the impact of these multiple factors and others influencing codon bias in plants.

## 2. Materials and methods

### 2.1. Sequence data

The genomic sequences and annotation data for 21 plant species were downloaded from Phytozome (http://www.phytozome.net).^21^ We analysed the sequences of 4 monocots [*Brachypodium distachyon* (BD), *O. sativa* (OS), *Sorghum bicolor* (SB) and *Zea mays* (ZM)], 15 dicots [*Arabidopsis lyrata* (AL)*, A. thaliana* (AT)*, Brassica rapa* (BR)*, Citrus clementina* (CC)*, Citrus sinensis* (CS)*, Eucalyptus grandis* (EG)*, Glycine max* (GM)*, Linum usitatissimum* (LU)*, Medicago truncatula* (MT)*, Populus trichocarpa* (PopT)*, Phaseolus vulgaris* (PV)*, Solanum lycopersicum* (SL)*, Solanum tuberosum* (ST)*, Thellungiella halophila* (TH) and *Vitis vinifer*a (VV)] and 2 mosses [*Physcomitrella patens* (PP) and *Selaginella moellendorffii* (SM)].

We used gff2sequence^22^ to identify coding sequences, proteins, introns and intergenic sequences. Coding sequences featuring non-canonical bases (other than A, C, G or T), missing stop codons or incomplete triplets were excluded. Finally, the longest splicing variant was chosen when multiple transcripts representing the same gene were annotated if not otherwise specified. Monocot coding sequences were divided into three subsets for analysis: (i) the entire genome, (ii) high G + C (HGC) content genes, >60% and (iii) low G + C (LGC) content genes, ≤60%.

### 2.2. Expression data

Gene expression data for Arabidopsis and rice were downloaded from the Plexdb database (http://www.plexdb.org/).^23^ We chose the expression atlases representing Arabidopsis dataset AT40 (http://www.plexdb.org/modules/PD_browse/experiment_browser.php?experiment=AT40) and rice dataset OS9 (http://www.plexdb.org/modules/PD_browse/experiment_browser.php?experiment=OS9). All the expression data were normalized by robust multi-array averaging (RMA). An expression value was calculated for each gene by averaging the replicates within each experiment and then computing a mean value over all the experiments in which the corresponding gene was expressed.^24^

### 2.3. Intergenic controlled coding sequences

For each gene, the first 100 two-fold and four-fold degenerate codons were used to create the coding sequence for analysis. Transcripts with fewer degenerate codons were excluded. The ICCSs were generated to estimate the influence of the background composition on coding sequence codon bias. Upstream and downstream sequences longer than 50 bp were extracted from the leading strand and joined together to form a set of concatenated intergenic sequences (CISs).

**Figure 1.**
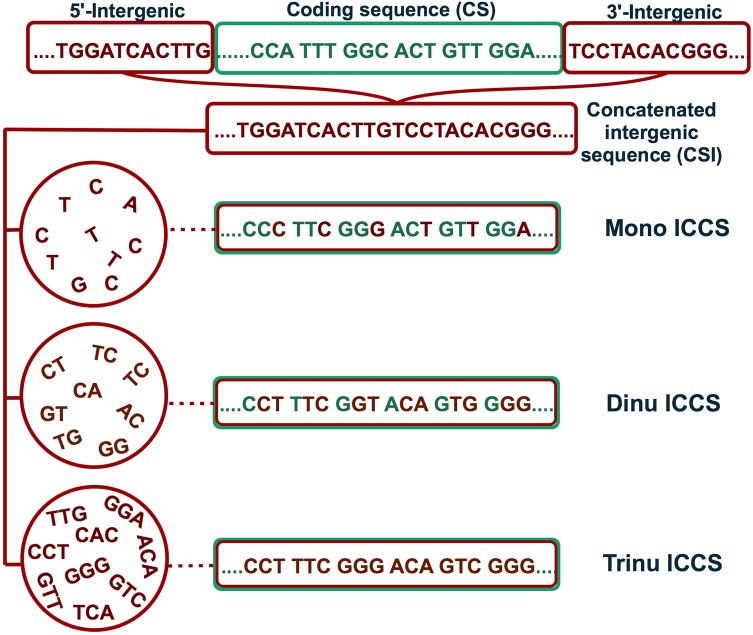
Method used for the construction of the mononucleotide ICCS (monoICCS), dinucleotide ICCS (dinuICCS) and trinucleotide ICCS (trinuICCS) data sets. This figure is available in black and white in print and in colour at *DNA Research* online.

Four different background controls were used to generate the ICCS datasets, beginning with the mononucleotide composition as originally used in the Hershberg and Petrov method.^19^ Briefly, a subsequence of 100 consecutive base pairs was randomly selected from the CIS, and the third base of each codon in the coding sequence was replaced with a nucleotide from this CIS subsequence (Fig. 1). This yielded a new data set called monoICCS. The intergenic dinucleotide composition was used to generate a second class of ICCS (dinuICCS) by choosing a random subsequence of 200 consecutive nucleotides from the CIS and picking the second base of each coding sequence codon randomly from within that subsequence. The adjacent base was then selected as the third codon position in the ICCS (Fig. 1). If the CIS was shorter than 100 bp, it was excluded from the monoICCS, and if it was shorter than 200 bp, it was excluded from the dinuICCS. Genes were also excluded from further analysis if any base in the CIS occurred fewer than four times.

Intergenic trinucleotide controlled coding sequences (trinuICCS) were produced by randomly picking the first dinucleotide for each coding sequence codon from within the CIS and selecting the adjacent nucleotide as the third base for the ICCS (Fig. 1). Finally, CDCB was estimated by randomly selecting an interrupted dinucleotide comprising the second base of each coding sequence codon and the first base of the subsequent codon. The intervening nucleotide was then selected as the third codon base in the CDCB intergenic controlled coding sequence (cdcbICCS). Genes in the trinuICCS and cdcbICCS data sets were excluded from further analysis if the corresponding dinucleotide appeared fewer than four times in the CIS.

To maintain the coding sequence amino acid structure, two-fold degenerate codons ending in A|G produced an ICCS ending in A when the corresponding intergenic position was A or T, and otherwise the ICCS ended in G. Similarly, two-fold degenerate codons ending in T|C produced an ICCS featuring T at the third codon position when the corresponding CIS nucleotide was A or T, and otherwise the ICCS ended in C.

### 2.4. Codon bias measurements

The effective number of codons (Nc)^25^ was used to estimate the overall codon bias for each gene in the CS and ICCS data sets. The Nc index gave higher values for less-biased genes (theoretical values between 21 and 61). To explore the contribution of each individual codon, relative synonymous codon usage (RSCU) values were computed for informative two-fold and four-fold degenerate codons (i.e. excluding methionine, tryptophan, stop codons, as well as three-fold and six-fold degenerate codons to avoid uneven nucleotide representation at the third position within the same codon family). RSCU values were calculated as the ratio of the observed and expected codon frequencies, i.e. the random use of all codons within a specific degenerate family.^26^

### 2.5. Signature of selection

For the 38 codons with two-fold or four-fold degeneracy, differences between the average RSCU values in the CS and ICCS data sets were calculated to highlight over-representation and under-representation in the coding sequences. For each codon *cod*, the deviation from background was calculated as follows:

$$\Delta \text{RSC}{\text{U}}^{\mathrm{cod}}=\frac{{\text{RSCU}}_{\mathrm{CS}}^{\mathrm{cod}}-{\text{RSCU}}_{\mathrm{ICCS}}^{\mathrm{cod}}}{\text{De}{\text{g}}^{\mathrm{cod}}}$$

where Deg^cod^ is the degeneracy of the codon family. The significance of highlighted differences was tested using a paired Wilcoxon test. The same statistical analysis was applied to highlight differences in the effective number of codons between the CS and ICCS datasets.

The association between the RSCU values of the CS and ICCS datasets and the gene expression levels was investigated in Arabidopsis and rice by sorting genes on the basis of their expression levels into 20 bins containing the same number of genes. The bin rank was then plotted against the average CS|ICCS RSCU value within the bin using either the intergenic or intron portion for the construction of the ICCS data set. In the latter case, all introns within the same gene were concatenated. In rice, this analysis was also carried out separately on the HGC and LGC gene sets.

Differences in codon bias between the CS and ICCS data sets were also calculated in several different portions of the coding sequences in a subset of plant species (AT, BR, OS, BD, PV, MT and SM). The unequal distribution of codon bias within individual transcripts is a well-known phenomenon that may cause different levels of deviation from the background.^27–29^ To investigate this phenomenon, we filtered coding sequences >1,500 bp in length and split them into three portions: the 5′ region comprising the first 501 nucleotides, the 3′ region the last 501 nucleotides and the middle region containing the remaining part of the coding sequence. The ΔRSCU value and its significance were calculated for each of the three portions.

The analysis was repeated with the Arabidopsis and rice ICCS data sets using intergenic regions that were masked (e.g. repetitive elements and pseudogenes were removed according to data retrieved from the TAIR and Phytozome websites) and trimmed by 200 bp at both ends. The differences between the CS and ICCS data sets in both species were also analysed separately on genes showing regular or alternative splicing. Finally, the impact of the intergenic distance was investigated by analysing two Arabidopsis and rice data sets, one featuring long intergenic sequences (>1,000 bp for AT and >2,200 bp for OS) and the other featuring short intergenic sequences (≤1,000 bp for AT and ≤2,200 bp for OS).

## 3. Results

### 3.1. Codon bias in the ICCSs

Mutational bias is one of the main forces affecting synonymous codon choice in plants.^30^ In theory, if no additional forces act on the coding sequences, the third base of each codon should reflect the background nucleotide frequency in the genome. However, the direction and strength of the codon bias should be investigated in the local genomic context to correct for intragenomic compositional heterogeneity. Several authors have reported the non-random usage of specific dinucleotides^31^ and trinucleotides^11,18^ in plant genomes, and thus, the analysis of mutational bias should also take into account these compositional units.

We used the composition of intergenic regions as a proxy for background bias according to the Hershberg and Petrov method.^19^ The portion of codon bias caused by background composition was measured by generating several ICCS data sets taking into account the mononucleotide, dinucleotide, trinucleotide and context-dependent intergenic composition of each plant species.

**Figure 2.**
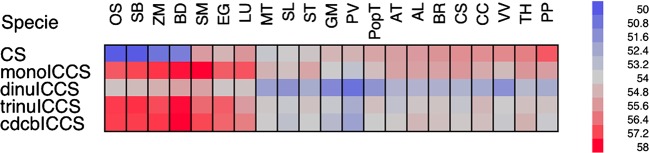
Heat map showing the average Nc values for the CS and ICCS data sets. This figure is available in black and white in print and in colour at *DNA Research* online.

We investigated the direction of mutational bias by analysing the codon bias among all the ICCS data sets. If the four nucleotides are equally and randomly distributed in the background, then there should be no codon bias in any of the ICCS data sets. In contrast, we observed the opposite trend, i.e. Nc values compatible with a multilevel background compositional bias. Interestingly, the lowest Nc values were found in the dinuICCS data set, revealing the strongest bias for all species (Fig. 2). The intensity of codon bias distinguished monocots and dicots. Whereas eudicots and particularly the legumes (*P. vulgaris*, *M. truncatula* and *G. max*) showed the highest ICCS codon bias at all levels, the opposite trend was observed for the monocots (rice, *Z. mays*, *S. bicolor* and *B. distachyon*) as shown in Fig. 2.

**Figure 3.**
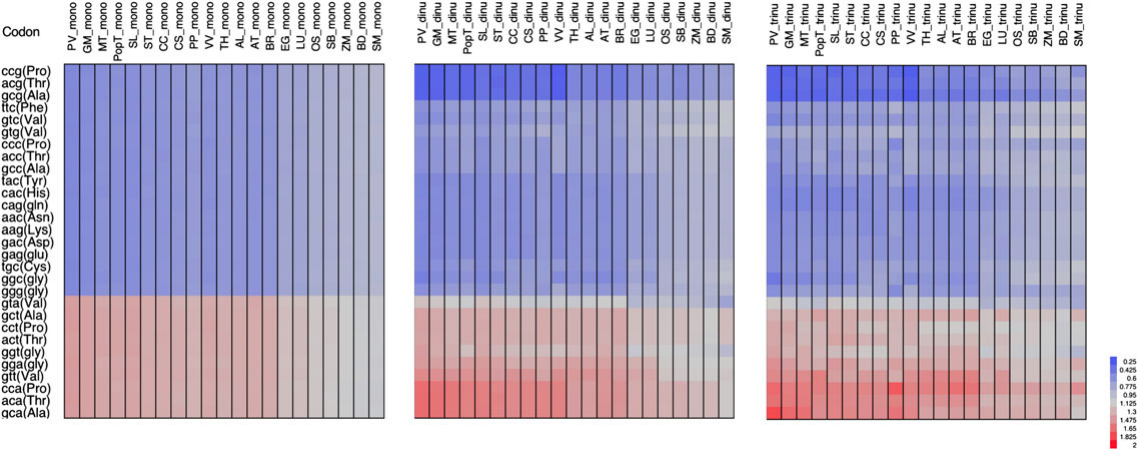
RSCU values calculated for the monoICCS, dinuICCS and trinuICCS data sets (only codons ending in G|C with two-fold degeneracy are shown). This figure is available in black and white in print and in colour at *DNA Research* online.

Nc values provide a snapshot of overall codon bias within genes but do not reveal the specific contributions of each codon. ICCS RSCU values were therefore calculated for all codons with two-fold or four-fold degeneracy (Fig. 3). We observed a significant over-representation of codons ending in A|T in the monoICCS data set of the eudicots, particularly *V. vinifera*, *Solanum* spp*.* and the legumes, and likewise in monocots and *S. moellendorffii* but to a much lesser extent.

A similar picture emerged from the analysis of the dinuICCS and trinuICCS RSCU values although several compositional signatures also emerged. The codons ending in A|T generally showed higher RSCU values at the expense of those ending in G|C, but the extent of the bias was variable. Indeed, codons ending in CG, AG and AC tended to be suppressed more strongly in the dinuICCS data set, e.g. these dinucleotides were under-represented in the intergenic regions of all the plants whereas codons ending in CA showed the opposite trend (Fig. 3). Only marginal differences were observed between the dinuICCS and trinuICCS data sets, indicating that the intergenic trinucleotide bias is predominantly caused by bias in the frequency of the underlying dinucleotides. Even less difference was observed between the cdcbICCS and trinuICCS datasets, indicating that CDCB does not reflect an analogous compositional pattern at a genomic level.

Differences in codon bias were distributed along the chromosomes of both monocots and dicots for all ICCS data sets (Supplementary Fig. S1), but spatial autocorrelation analysis failed to detect a significant effect on the ICCS RSCU values (Supplementary Fig. S2).^32^

### 3.2. Codon bias in the coding sequences

If synonymous codon choice solely reflects the distribution of nucleotides in the genomic background, there should be no differences in Nc value between the CS and ICCS data sets. However, our results revealed significant differences (Wilcoxon paired sample test) between these data sets, although the direction and extent of divergence were species dependent. Coding sequences were more biased in monocots than in dicots (Fig. 2) in sharp contrast to the comparison of ICCS data sets. Interestingly, the coding sequences were even less biased than the corresponding ICCSs in some species (i.e. GM and PV), with the trend most noticeable when comparing the CS and dinuICCS data sets. These data suggest that additional forces shape the coding sequences and oppose mutational bias, e.g. resulting in G|C enrichment despite background A|T enrichment.

**Figure 4.**
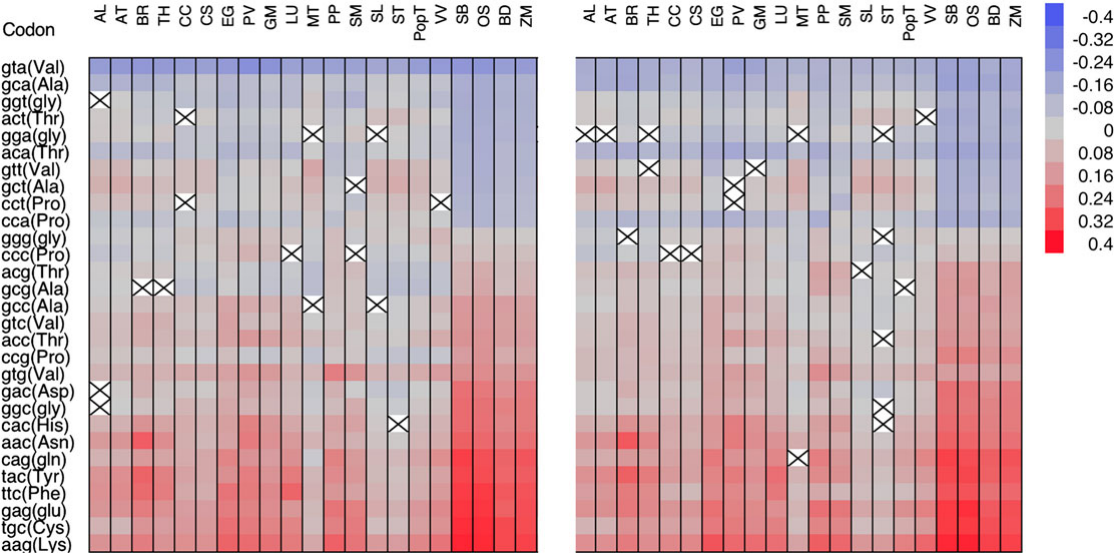
Calculation of ΔRSCU values as the standardized differences between the CS RSCU and the monoICCS (right) and the dinuICCS RSCUs (left). Black crosses in white squares show insignificant differences between the data sets. This figure is available in black and white in print and in colour at *DNA Research* online.

Differences in RSCU values between the CS and ICCS data sets allowed us to focus on codons whose frequency cannot be explained by background bias alone. Comparisons between the CS data set and the four ICCS data sets led to similar results (Fig. 4 and Supplementary Fig. S3). All codons ending in A|T were under-represented in the coding sequences, with GTA showing the greatest depletion. In contrast, several codons ending in G|C were over-represented in the coding sequence, with certain species-dependent exceptions. Codons CCC and GGG were suppressed in many species, together with codons CCG, GCG and ACG. The under-representation of these codons together with GTA was more striking when comparing the CS and monoICCS data sets and less pronounced when comparing the CS and dinuICCS data sets, suggesting that such a bias in the coding sequences in part reflects the general suppression of the TA|CG dinucleotide in the intergenic regions (Fig. 3). Some differences between the CS and ICCS data sets were also taxon dependent, e.g. the preference for G|C at the third codon position was more apparent in monocots than in eudicots, particularly *M. truncatula, V. vinifera* and *Solanum* spp., where there was little evidence for preference (Fig. 4).

An unequal distribution of codon bias within individual transcripts has been observed in many species^27–29^ so we also studied the differences between the CS and ICCS data sets in three different portions of the coding sequences. Accordingly, we observed a variation in ΔRSCU values along the transcript, with the 5′ regions featuring the greatest differences (Supplementary Fig. S4).

The influence of recurrent motifs within the intergenic regions was tested by using a hard-masked version of the genome and trimming the intergenic portions by 200 bp at each end. Additionally, the impact of intergenic length on the ΔRSCU value was investigated by splitting the original data set into two subsets representing short and long intergenic sequences. In both cases, no differences were observed compared with the original data set (Supplementary Figs S5 and S6).

Finally, a recent report has suggested that rice genes experience differential selection depending on whether they undergo regular invariant splicing or alternative splicing.^33^ We therefore calculated the differences between the CS and ICCS data sets representing each set of genes separately and observed a strong correlation between the results of each analysis (Supplementary Fig. S7).

### 3.3. Correlation between codon bias and gene expression in Arabidopsis and rice

**Figure 5.**
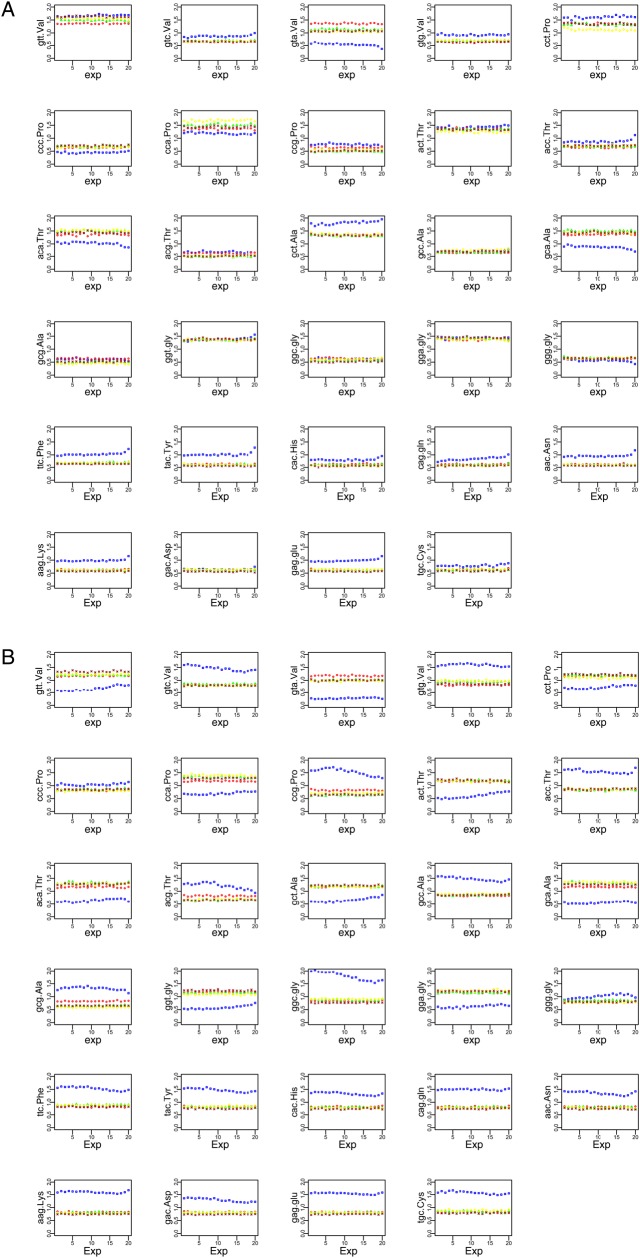
RSCU values of (A) Arabidopsis and (B) rice genes after splitting the data sets into 20 expression bins. Intergenic sequences were used for the construction of the ICCS data sets. Symbol codes: square, CS; circle, monoICCS; triangle, dinuICCS; diamond, trinuICCS; cross, cdcbICCS. This figure is available in black and white in print and in colour at *DNA Research* online.

Codon bias in the species we investigated was not fully explained by background compositional differences so we investigated the influence of gene expression on synonymous codon usage in the CS and ICCS data sets. Codons that are translated more rapidly or accurately should be preferentially found in the coding sequences of genes, particularly those expressed at high levels. We chose Arabidopsis and rice as representative dicot and monocot species and assigned genes to 20 bins based on expression levels, and then mapped the RSCU values of the CS and the ICCS data sets onto these bins (Fig. 5). This strategy should also reveal additional phenomena other than translational selection. For example, a codon whose frequency is positively associated with expression level may be under selection to optimize translation efficiency and/or accuracy. However, if genes with comparable expression levels share the same composition as the intergenic sequences, this would also generate variation in their ICCS RSCU values. Furthermore, if the RSCU values in the CS and ICCS data sets differ regardless of the expression level, then forces other than the translational selection are likely to be responsible.

We found that two-fold degenerate codons ending in G|C are more frequent in the coding sequences of rice and Arabidopsis, although in the latter case the aspartic acid codon GAC was an exception. There was no association between expression level and the RSCU values of the ICCSs, suggesting that expression-dependent variation in codon bias does not reflect differences in the background composition of the Arabidopsis genome. Weaker trends were observed in Arabidopsis when the four-fold degenerate codons were analysed both in terms of deviation from the background and variation with expression. Indeed, the frequency of many codons mirrored the background composition of the entire set of bins, so that the curves generated by the CS and ICCS data sets could be superimposed. However, there was a positive association between the RSCU values and expression levels of codons ending in C, and the opposite trend was observed for codons ending in A. Interestingly, the frequency of several codons ending in T was positively associated with the expression level, particularly the alanine codon CGT.

A more complex picture emerged from the analysis of rice genes. The correlation between RSCU values and gene expression was non-monotonic for codons with two-fold degeneracy, although as in Arabidopsis the codons ending in G|C were still found more frequently in the coding sequences. However, an initial reduction in the frequency of such codons was complemented by an increase in RSCU values at higher expression levels. If we exclude the effect of the background composition, which again does not vary with the expression level, such non-monotonic trends may reflect the coexistence of contrasting forces whose strength could be dependent on the expression level. Alternatively, the different expression bins in rice may be populated with genes that do not experience the same selective pressure. Indeed, it is well known that monocots have two classes of genes that differ in G + C content.^13^ For this reason, the above analysis was repeated focusing specifically on LGC and HGC genes. The LGC genes accounted for most of the dataset and mirrored the overall trends discussed above, but the HGC genes showed a general positive association between the expression level and the RSCU values of two-fold degenerate codons ending in G|C.

In rice, four-fold degenerate codons ending in G|C were always used more frequently in the coding sequence than the ICCS, whereas the opposite trend was observed for codons ending in A|T. Furthermore, codons ending in G|C were generally used less frequently in strongly expressed genes, whereas there was a positive association between codons ending in T and expression level. As previously reported, such trends are often non-monotonic, either disappearing or changing direction when HGC and LGC genes are analysed separately (Supplementary Fig. S8).

The positive association between the RSCU value and expression level of codons ending in T is reminiscent of transcription-associated mutational bias (TAMB), a well-known repair system that increases bias towards G|T rather than C|A in the pre-mRNA sequence.^34^ For this reason, we repeated our analysis by constructing a new set of ICCSs using intron sequences as a proxy for the background composition. If codons ending in T become more frequent in strongly expressed genes possibly due to TAMB, the same trend should be observed in intron-corrected coding sequences. Our results revealed no such association (Supplementary Fig. S9), indicating that TAMB is not responsible for the positive association between gene expression level and the frequency of codons ending in T.

## 4. Discussion

Several ICCS data sets were generated to investigate whether codon bias in the coding sequence reflected the genomic background nucleotide composition of the plant species included in this study. Four ICCS data sets constructed using different strategies were analysed to determine the effective number of codons and RSCU.

### 4.1. Background composition

The RSCU values of the monoICCS data set revealed the over-representation of A|T compared with G|C although to a different extent in each species. The legumes and solanaceous species showed the greatest difference in the representation of A|T and G|C, whereas there was a weaker distinction in the monocot species, and the brassicas showed intermediate values. G|C to A|T mutations may be more frequent in Arabidopsis, leading to A|T enrichment in the genome.^35^ The deamination of methylated cytosine residues at CG dinucleotide motifs and the UV-induced mutagenesis of dipyrimidines (CC and TC) were proposed as the main mechanisms to explain this phenomenon.^35^ Several factors may account for the observed differences in the background nucleotide composition of plants. In monocots, the vertical leaf orientation, protective basal sheath and concealed apical meristem make the interception of solar radiation less efficient,^36^ thus reducing the prevalence of AT enrichment induced by UV light. Domestication may also contribute to this bias, because A|T pairs contain seven nitrogen atoms compared with the eight present in G|C pairs, which may drive A|T enrichment in non-cultivated plants.^37^ However, the cluster analysis of ICCS RSCU values revealed a pattern that is consistent with the genomic composition of plants solely depending on phylogeny (Supplementary Fig. S10) rather than the contrast between wild and cultivated plants.

A strong dinucleotide compositional bias was evident in the ICCS data set of all species as shown by the higher levels of codon bias (i.e. lower Nc values) in the dinuICCS data set compared with the others (Fig. 2). We found that codons ending in CG were among the most under-represented in the dinuICCS data set, whereas codons ending in CA (the reverse complement of the C deamination product in CG) were among the most over-represented, as recently reported in Arabidopsis and rice.^31^ Codons ending in CC and TC were also suppressed in the dinuICCS data set, confirming the under-representation of these dinucleotides in non-coding sequences.^18^

### 4.2. Differences between the CS and ICCS data sets

The compositional pattern of several genomes has been investigated at different levels, including the whole genome and the genic portion.^18^ However, to the best of our knowledge, this is the first time that coding and adjacent non-coding sequences have been directly compared in terms of the mononucleotide, dinucleotide and trinucleotide frequencies as well as CDCB. Paired Wilcoxon tests generally revealed significant differences in codon usage between the CS and ICCS data sets in terms of both the Nc and RSCU values. The observed differences were similar in magnitude when comparing the CS data set with all four methods for the construction of ICCS data sets. This finding validates the Hershberg and Petrov method for the identification of optimal codons in plants even though this only considers the mononucleotide composition of the background sequences. The optimal codon data sets calculated using this method could be almost perfectly superimposed over the data sets generated using the other three methods (Supplementary Fig. S11). The comparison between CS and ICCS allowed us to distinguish codon bias caused by genomic compositional features from codon bias related to the expressed portion of the genome.

The comparison of CS and ICCS data sets suggested that there was enrichment for codons ending with G or C particularly in monocots, although among the codons ending in G|C the frequency of the complementary codons GGG and CCC was close to the genomic background. This suggests the existence of selection against codons that promote the formation of complex mRNA tertiary structures, and the prevalence of this phenomenon in monocots with their higher overall G + C content may emphasize such an underlying mechanism.

Codons ACG, CCG and GCG were marginally over-represented in the coding sequences of some species and under-represented in others. This supports the observation that the CG dinucleotide is suppressed, in part due to deleterious methylation/deamination events. However, our analysis suggested that this phenomenon reflects an underlying genomic tendency, because the effects were not seen when comparing the CS and dinuICCS data sets (Fig. 4).

Finally, the general suppression of codon GTA was one of the most conserved features among the plant species we investigated. This is not surprising, because the codon ends with dinucleotide TA, which is known to be suppressed in the coding sequences of several plant species^18,31^ possibly to discourage insertion events that target the TA dinucleotide,^38^ to reduce the likelihood of mutations leading to stop codons and to prevent attacks by TA-specific RNases.^39^ The less striking divergence between the CS and dinuICCS data sets in terms of GTA preference suggests that TA suppression is also a general genomic signature (Fig. 3).

We also demonstrated that the differences between the CS and ICCS vary along the transcript (Supplementary Fig. S4), although the background composition cannot explain the observed codon bias in all three portions of the coding sequence that were analysed individually. Differences between CS and ICCS were stronger in the first portion of the transcript in agreement with previous studies reporting stronger selection in the proximal region of the transcript.^40^

### 4.3. Mutational bias in coding sequences

The background nucleotide pattern alone cannot explain the observed codon bias in the coding sequences, so additional forces must be involved. Indeed, modestly frequent amino acids (or under-represented amino acids in the proteins encoded by strongly expressed genes) may be under weaker selection, and for this reason, their codons may better tolerate changes driven by mutational bias. For example, histidine and cysteine are among the less abundant amino acids in the Arabidopsis proteome, and there is a significant negative correlation between their frequency and the expression level of the corresponding genes (Supplementary Table S1). The frequency of codons CAC (His) and TGC (Cys) in Arabidopsis is similar in the CS and ICCS data sets (Fig. 5). The codons GAC (Asp), GGC (Gly) and GCC (Ala) also revealed biases that mirrored the background composition. Interestingly, these codons feature the generic sequence CNG that has been proposed as the minimal building block for globular proteins and, for this reason, may represent the core of the primitive genetic code.^41^ Such a trend may therefore reflect the more prolonged effect of mutational bias on the most ancient codons. In contrast, there appeared to be little mutational bias in monocots, where other factors increase the frequency codons ending in G|C.

### 4.4. Translational selection and mRNA stability

Genes were assigned to 20 expression bins whose average RSCU values were plotted for the CS data set and all four ICCS data sets for Arabidopsis and rice, representing the eudicots and monocots, respectively (Fig. 5). ICCS RSCU values were not associated with the expression level, suggesting that RSCU variation within coding sequences cannot be explained by the background composition.

In Arabidopsis, we observed a positive correlation between gene expression level and the frequency of optimal codons^42,43^ ending in G|C, mainly for genes assigned to the last few expression bins, underlining the marginal effect of translational selection in this species.^42^ A more complex scenario emerged in rice, where non-monotonic trends were observed for codons ending in G|C, i.e. a reduction in frequency in the first expression bins changing to an increase in frequency in the last few. This behaviour may be typical of G + C rich monocot genomes and may indicate a compromise between the advantage of using optimal codons and the avoidance of tightly packed mRNA tertiary structures. Indeed a significant negative correlation was found between the expression level and both the coding sequence length and the G + C content of rice genes.^44^ Taken together, these data suggest that weakly expressed genes are longer and have a lower G + C content at the third codon position than strongly expressed genes. Longer transcripts are more likely to form strongly packed mRNA tertiary structures if they are enriched in optimal codons ending in G|C, so the accumulation of such codons may be counterselected in genes expressed at low or moderate levels. Such a trend would diminish in shorter genes expressed at high levels. This hypothesis was confirmed by analysing HGC and LGC rice genes separately. We found that short HGC genes were generally characterized by a monotonic positive association between the expression level and the frequency of optimal codons ending in G|C.

### 4.5. Additional factors

TAMB is a well-known DNA repair system that influences the composition of primary transcripts (both exons and introns) by increasing the prevalence of G|T over C|A. Despite this general G + C enrichment, we observed the enrichment of four-fold degenerate codons ending in T, suggesting that TAMB may affect codon bias in Arabidopsis and rice, so we repeated our analysis using introns rather than intergenic DNA as an index of background composition. However, we found no differences in the frequency of codons ending in T when we used introns rather than intergenic DNA (Supplementary Fig. S9).

The increasing G + C content of plant genomes may have been driven by G + C-biased gene conversion (gBGC) during double-strand break repair followed by a recombination event.^45^ This phenomenon is accompanied by the correction of eventual mismatches between two paired DNA strands featuring high-sequence similarity, with such a correction being biased towards the placement of either G or C. Given that gBGC is not restricted to genes, the differences in codon bias between the CS and ICCS data sets would not highlight gBGC events. However, synonymous substitutions caused by gBGC are more likely to be fixed in the coding sequence, because codons ending in G|C are known to mirror the most abundant tRNAs in most plant species. There is also evidence that recombination in plant genomes occurs mainly within genes.^46^

Our data provided several lines of evidence supporting the occurrence of gBGC in plants. Indeed if gBGC rather than selection is considered to be the main source of G|C enrichment at the third codon position, such an effect should be evident in all the transcripts regardless of the expression level. As shown in Fig. 5, this was the case for the majority of the codons and expression bins in both Arabidopsis and rice, with the latter featuring wider divergence from the background in accordance with previous reports of gBGC in grasses.^47^

## Supplementary data

Supplementary data are available at www.dnaresearch.oxfordjournals.org

## Funding

We would like to thank the Università di Sassari for funding. Funding to pay the Open Access publication charges for this article was also provided by the Università di Sassari.

## Supplementary Material

Supplementary Data
