# Supplementary material for: New insights into the interplay between codon bias determinants in plants
Source: DNA Res. 2015 Nov 5;22(6):461–70. doi: 10.1093/dnares/dsv027 (PMC4675714; doi:10.1093/dnares/dsv027)
Supplement: Supplementary Data [file supp_22_6_461__index.html]

New insights into the interplay between codon bias determinants in plants — Supplementary Data 

# New insights into the interplay between codon bias determinants in plants

## Supplementary Data

Supplementary Data

- Supplementary Data - Doc file
- Supplementary Figure 1 - tiff file
- Supplementary Figure 2 - jpg file
- Supplementary Figure 3 - tiff file
- Supplementary Figure 4 - tiff file
- Supplementary Figure 5 - tiff file
- Supplementary Figure 6 - tiff file
- Supplementary Figure 7 - tiff file
- Supplementary Figure 8 - tiff file
- Supplementary Figure 9 - tiff file
- Supplementary Figure 10 - tiff file
- Supplementary Figure 11 - tiff file
- Supplementary Table 1 - pdf file
