# Supplementary material for: New insights into the interplay between codon bias determinants in plants
Source: DNA Res. 2015 Nov 5;22(6):461–70. doi: 10.1093/dnares/dsv027 (PMC4675714; doi:10.1093/dnares/dsv027)
Supplement: Supplementary Data [file supp_dsv027_dsv027supp.doc]

**Figure S1:** Variation in the extent of TTC relative synonymous codon bias along chromosome 1 of *Arabidopsis thaliana* (AT), *Oryza sativa* (OS) and *Homo sapiens* (HS). The plots were generated by averaging the RSCU values of the CS/ICCS datasets within sliding windows containing genes ordered by chromosome position (x = position, y = TTC RSCU, window size = 100 genes, window step = 10 genes). Colour codes: blue = CS, red = monoICCS, yellow = dinuICCS, brown = trinuICCS).

**Figure S2:** Spatial autocorrelation analysis for chromosome 1 of *Arabidopsis thaliana* (AT), *Oryza sativa* (OS) and *Homo sapiens* (HS). Moran indices were calculated for the (A) CS, (B) monoICCS, (C) dinuICCS, and (D) trinuICCS datasets.

**Figure S3**: Calculation of ΔRSCU values as the standardized differences between the CS RSCU and the monoICCS, dinuICCS, trinuICCS and cdcbICCS RSCUs. Black crosses in white squares indicate insignificant differences between the datasets. Heat maps for monoICCS and dinuICCS are represented for comparison.

**Figure S4:** Differences between the RSCU values of CS and ICCS (mono, dinu and trinu) in three portions of the coding sequences. 5’ = first 501 nucleotides, 3’ = last 501 nucleotides, Mid = coding sequence after trimming 5’ and 3’ portions. AT = Arabidopsis thaliana, BR = Brassica rapa, OS = Oryza sativa, BD = Brachipodium distachyon, PV = Faseulus vulgaris, MT= Medicaco truncatula, SM = Selaginella moellendorffii.

Figure S5: Scatterplot depicting the correlations between the ΔRSCU values calculated on the unmasked genomes and masked genomes for *Arabidopsis thaliana* and *Oryza sativa*. In order to calculate ICCS RSCU (mono, dinu and trinu) masked intergenic sequences were also trimmed of 200 nucleotides at their ends to account for the potential occurrence of recurrent motifs (e.g. TATA box, proximal promoters, etc.)

Figure S6: Scatterplot depicting the correlation between CS-ICCS (mono, dinu and trinu) ΔRSCU values calculated on two gene subsets featuring short and long intergenic regions for *Arabidopsis thaliana* and *Oryza sativa*.

Figure S7: Scatterplot depicting the correlation between CS-ICCS (mono, dinu and trinu) ΔRSCU values calculated on two gene subsets featuring alternative spliced (AS) and not alternative spliced (NAS) genes for *Arabidopsis thaliana* and *Oryza sativa*.

**Figure S8:** RSCU values of (a) LGC and (b) HGC rice genes after splitting the datasets into 20 expression bins. The intergenic sequences were used for the construction of the ICCS datasets. Colour codes: blue = CS, red = monoICCS, green = dinuICCS, yellow= trinuICCS, brown = cdcbICCS.

**Figure S9:** RSCU values for (a) Arabidopsis and (b) rice genes after splitting the datasets into 20 expression bins. Intron sequences were used for the construction of the ICCS datasets. Colour codes: blue = CS, red = monoICCS, green = dinuICCS, yellow = trinuICCS, brown = cdcbICCS.

**Figure S10:** Cluster analysis of (a) monoICCS, (b) dinuICCS and (c) trinuICCS RSCU values.

**Figure S11:** Venn plots showing the optimal codons calculated using the Hershberg and Petrov method ^19^ with four differently-constructed ICCS datasets representing genes from (a) Arabidopsis and (b) rice.
