# Supplementary figures and images for: New insights into the interplay between codon bias determinants in plants
Source: DNA Res. 2015 Nov 5;22(6):461–70. doi: 10.1093/dnares/dsv027 (PMC4675714; doi:10.1093/dnares/dsv027)

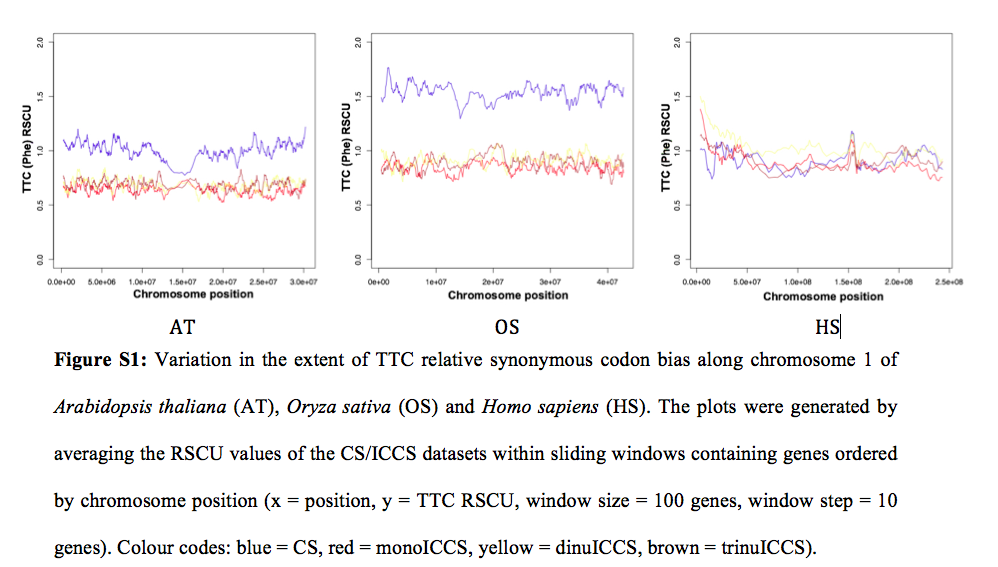

Supplement: Supplementary Data [file supp_dsv027_dsv027supp_fig1.tif]

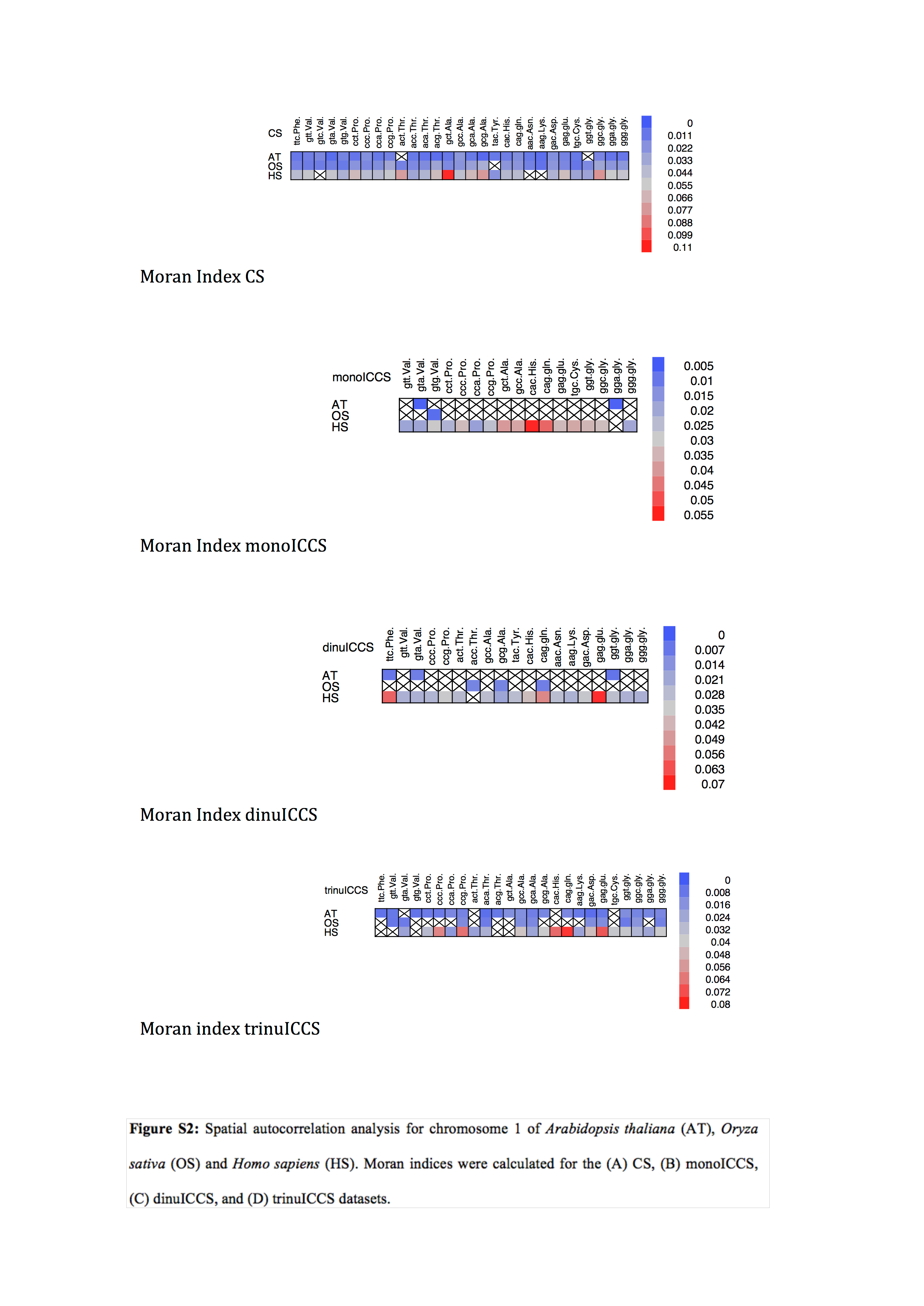

Supplement: Supplementary Data [file supp_dsv027_dsv027supp_fig2.jpg]

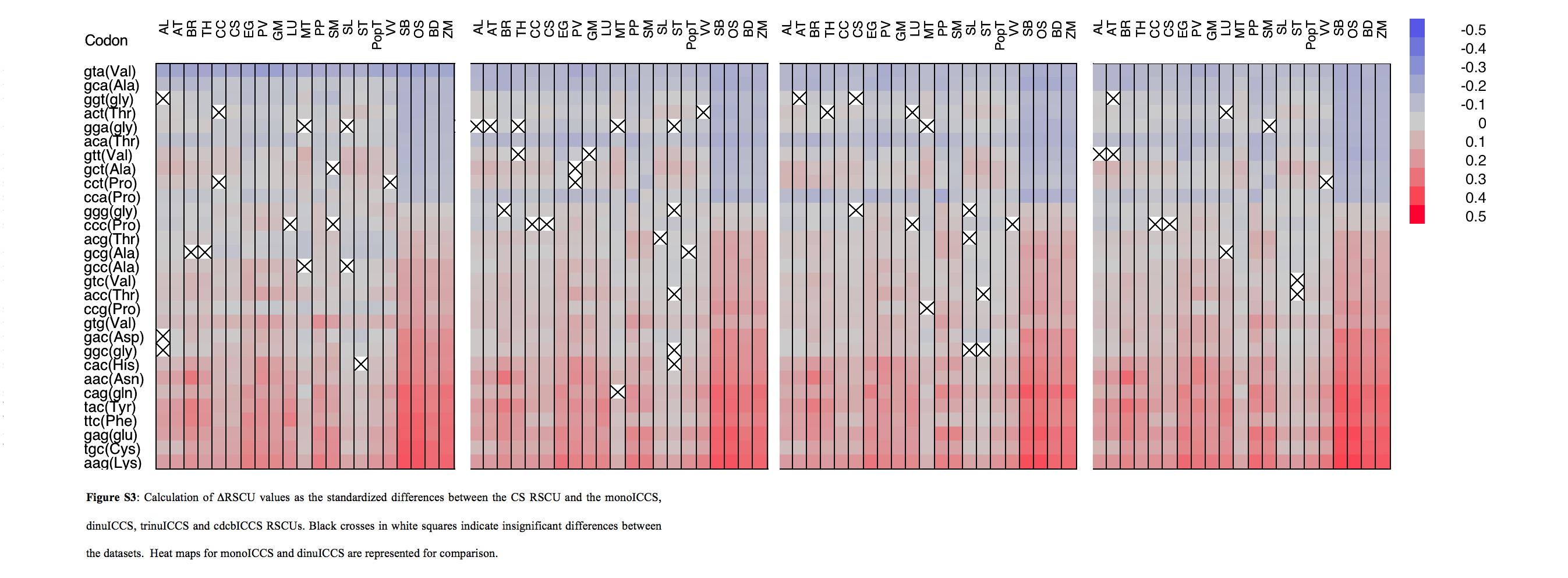

Supplement: Supplementary Data [file supp_dsv027_dsv027supp_fig3.tif]

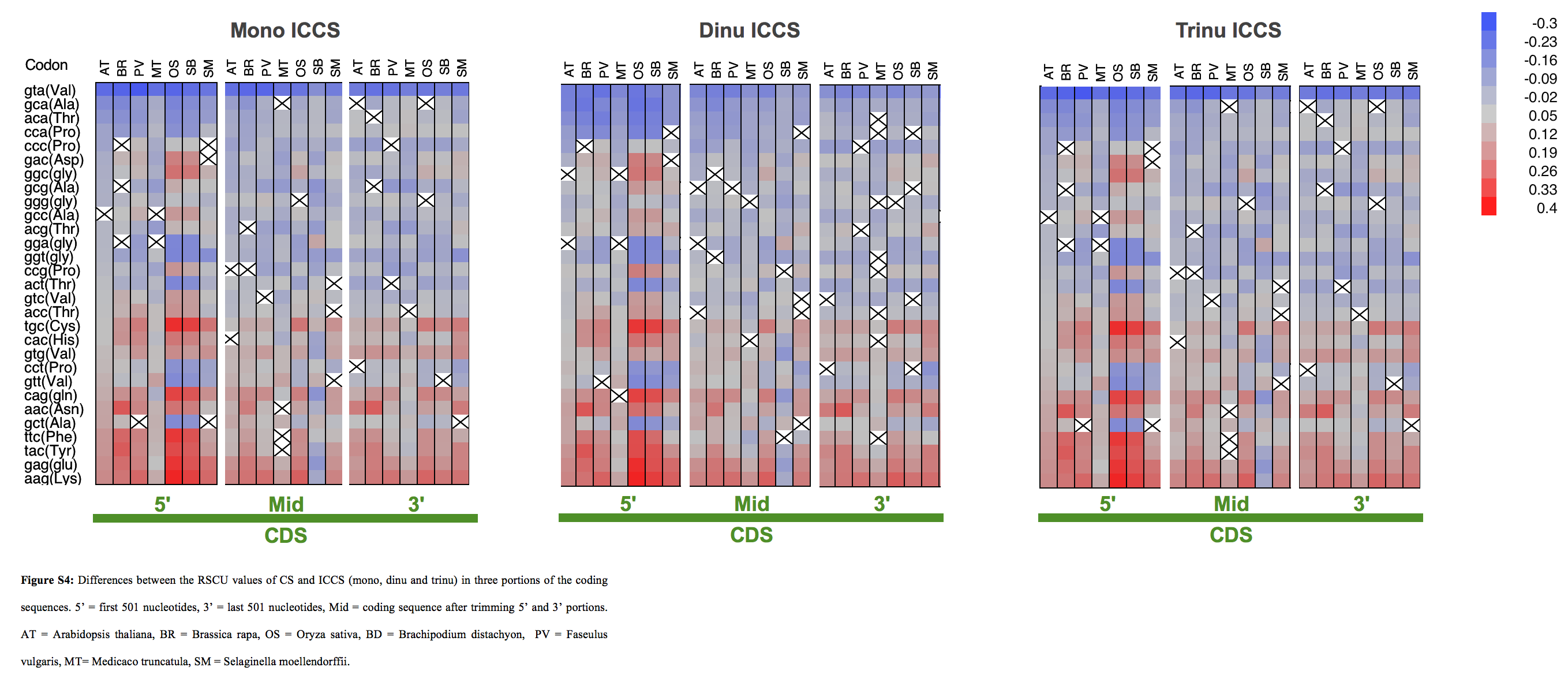

Supplement: Supplementary Data [file supp_dsv027_dsv027supp_fig4.tif]

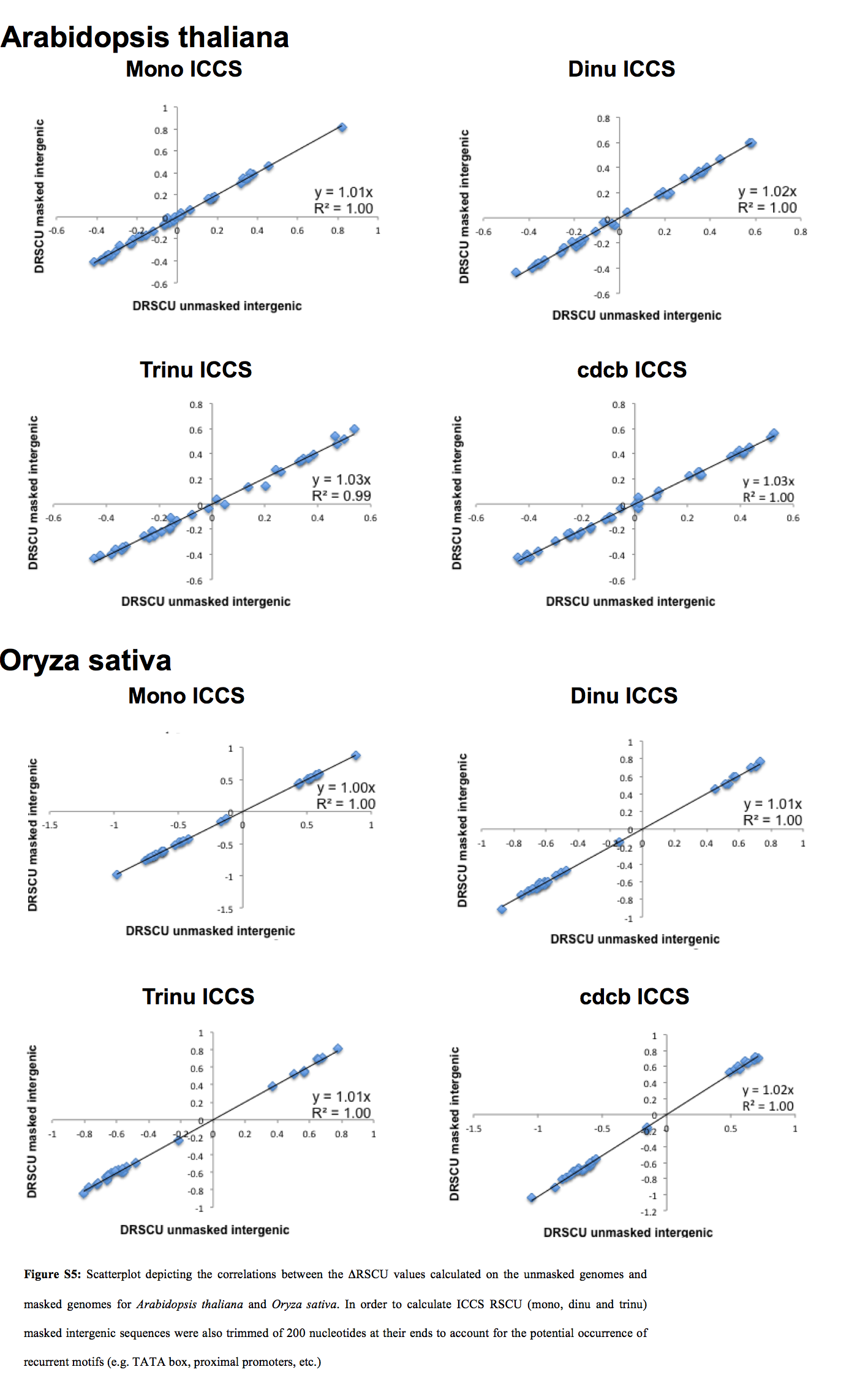

Supplement: Supplementary Data [file supp_dsv027_dsv027supp_fig5.tif]

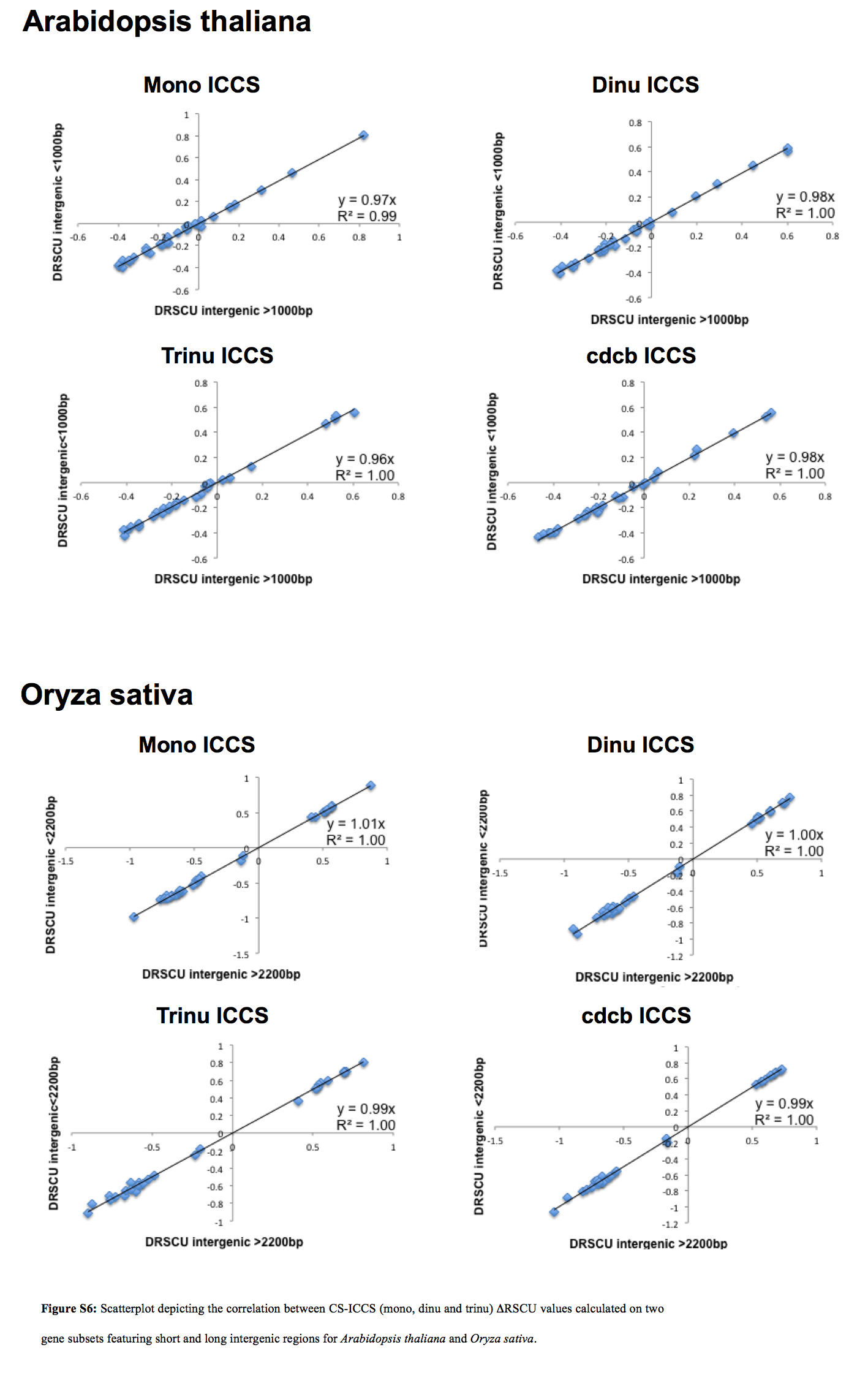

Supplement: Supplementary Data [file supp_dsv027_dsv027supp_fig6.tif]

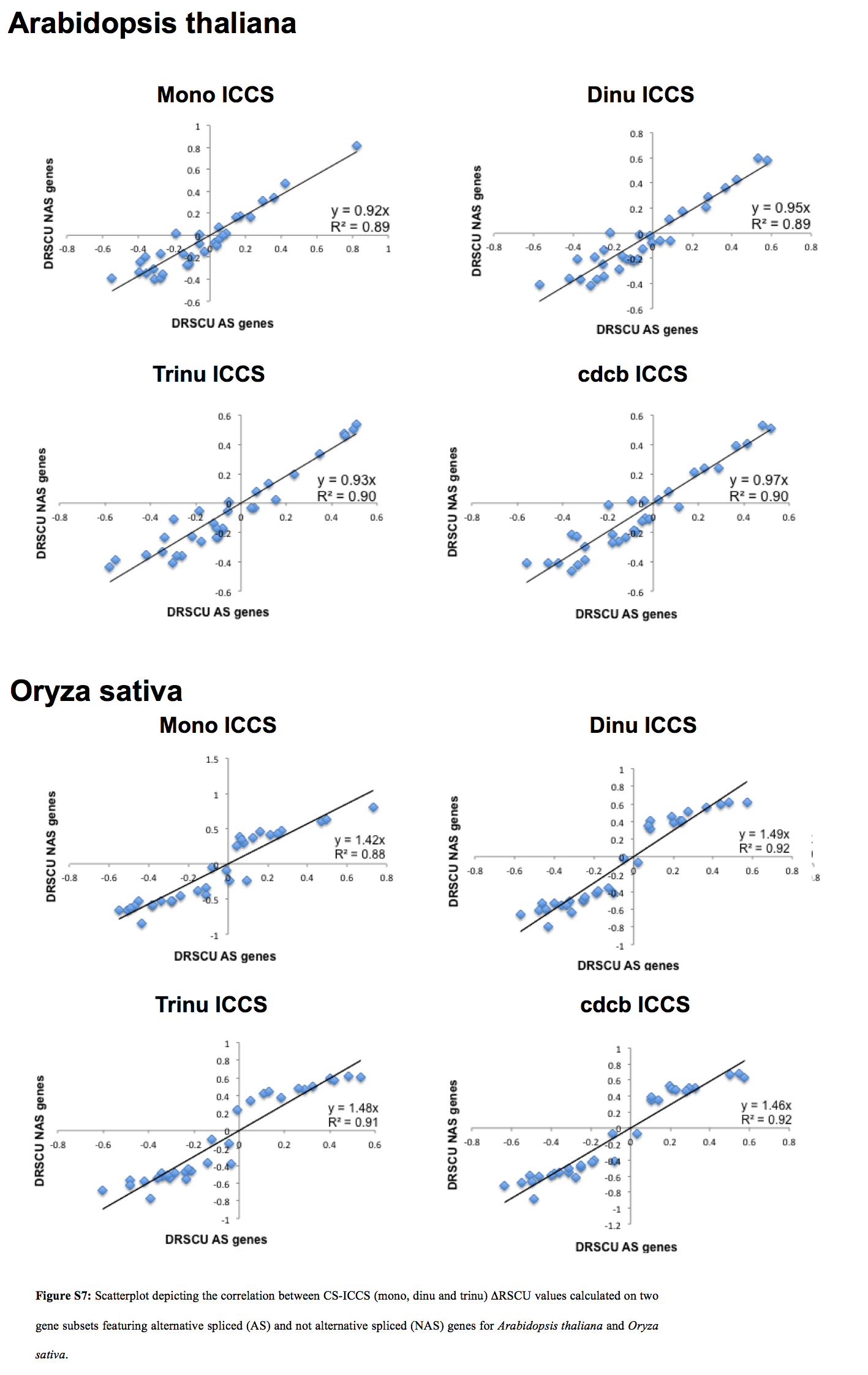

Supplement: Supplementary Data [file supp_dsv027_dsv027supp_fig7.tif]

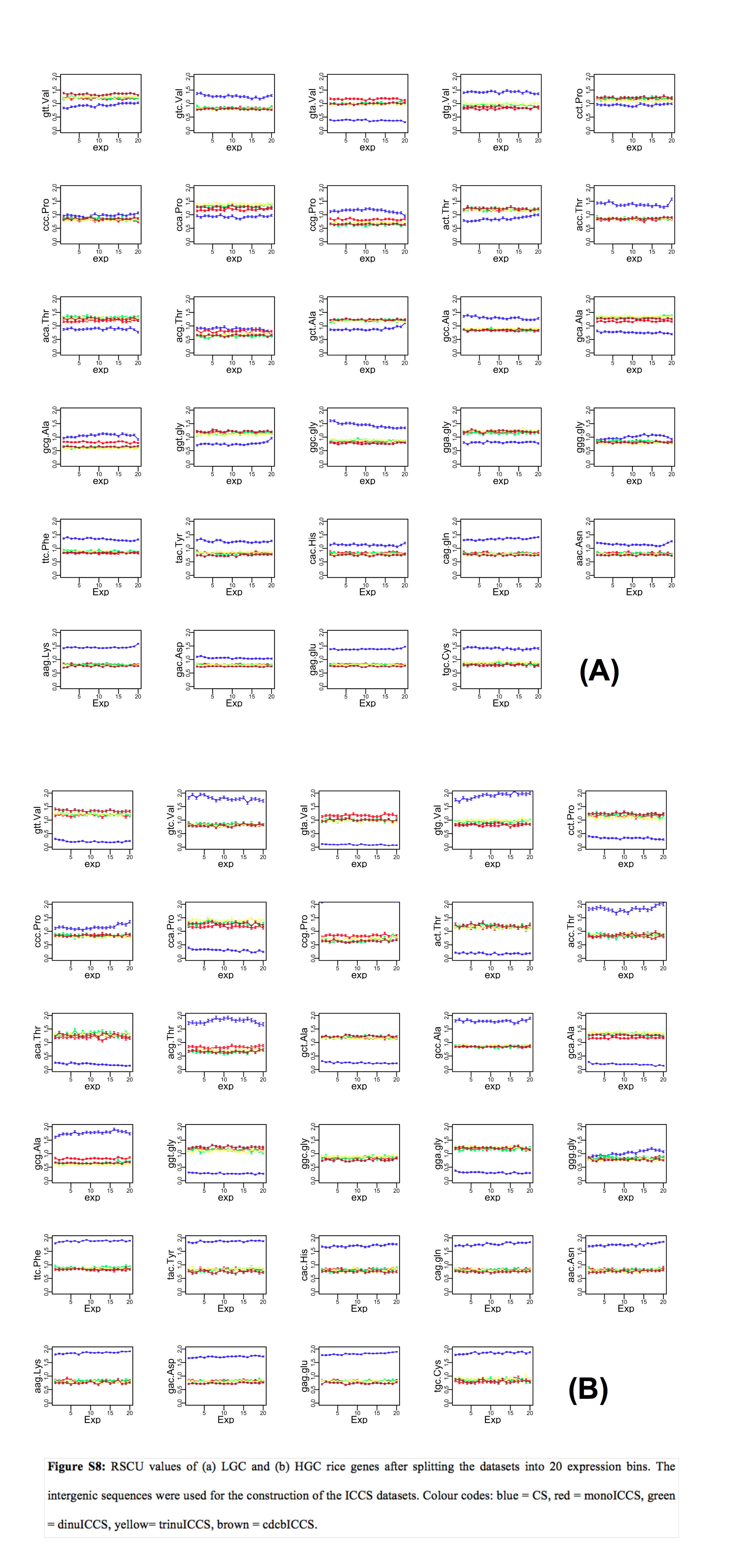

Supplement: Supplementary Data [file supp_dsv027_dsv027supp_fig8.tif]

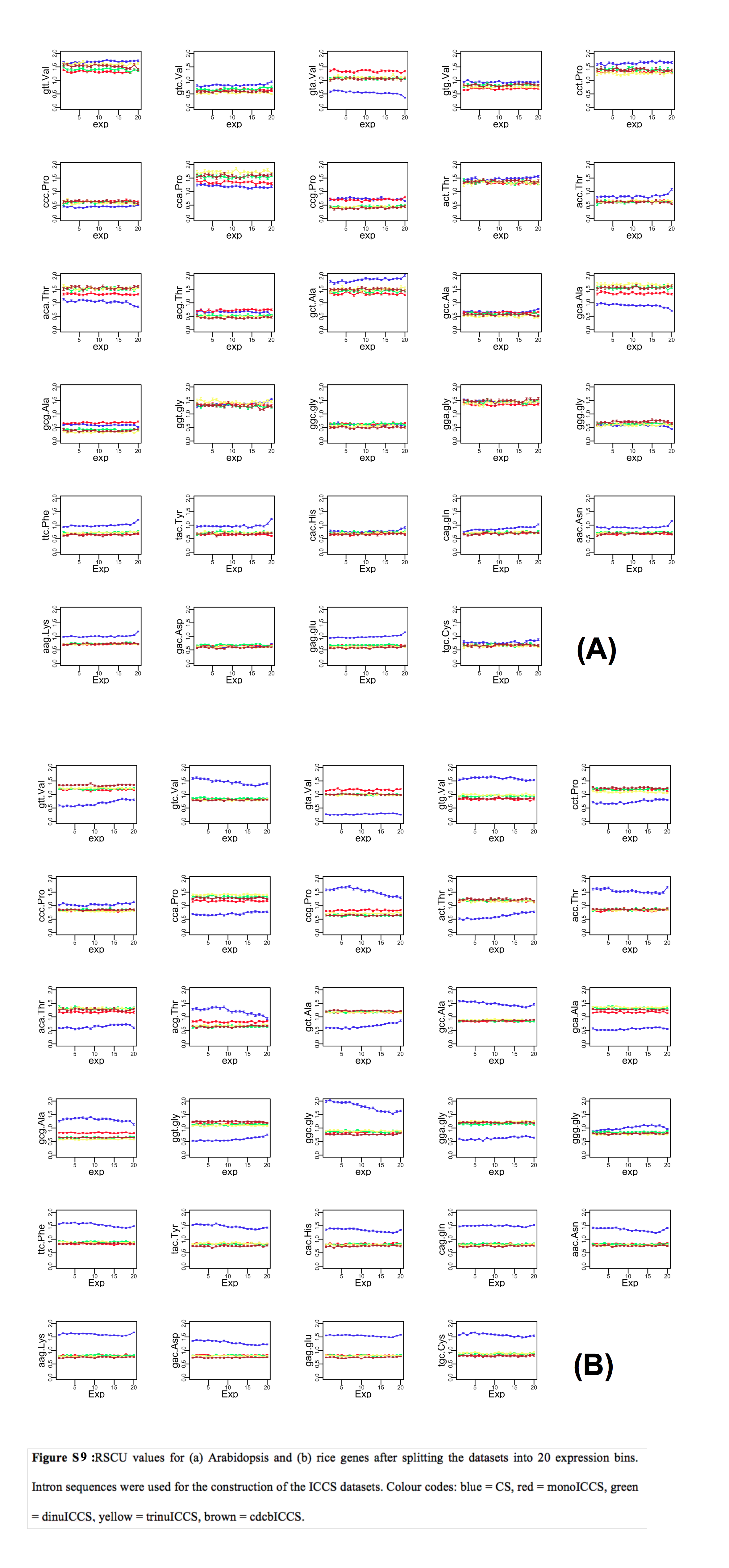

Supplement: Supplementary Data [file supp_dsv027_dsv027supp_fig9.tif]

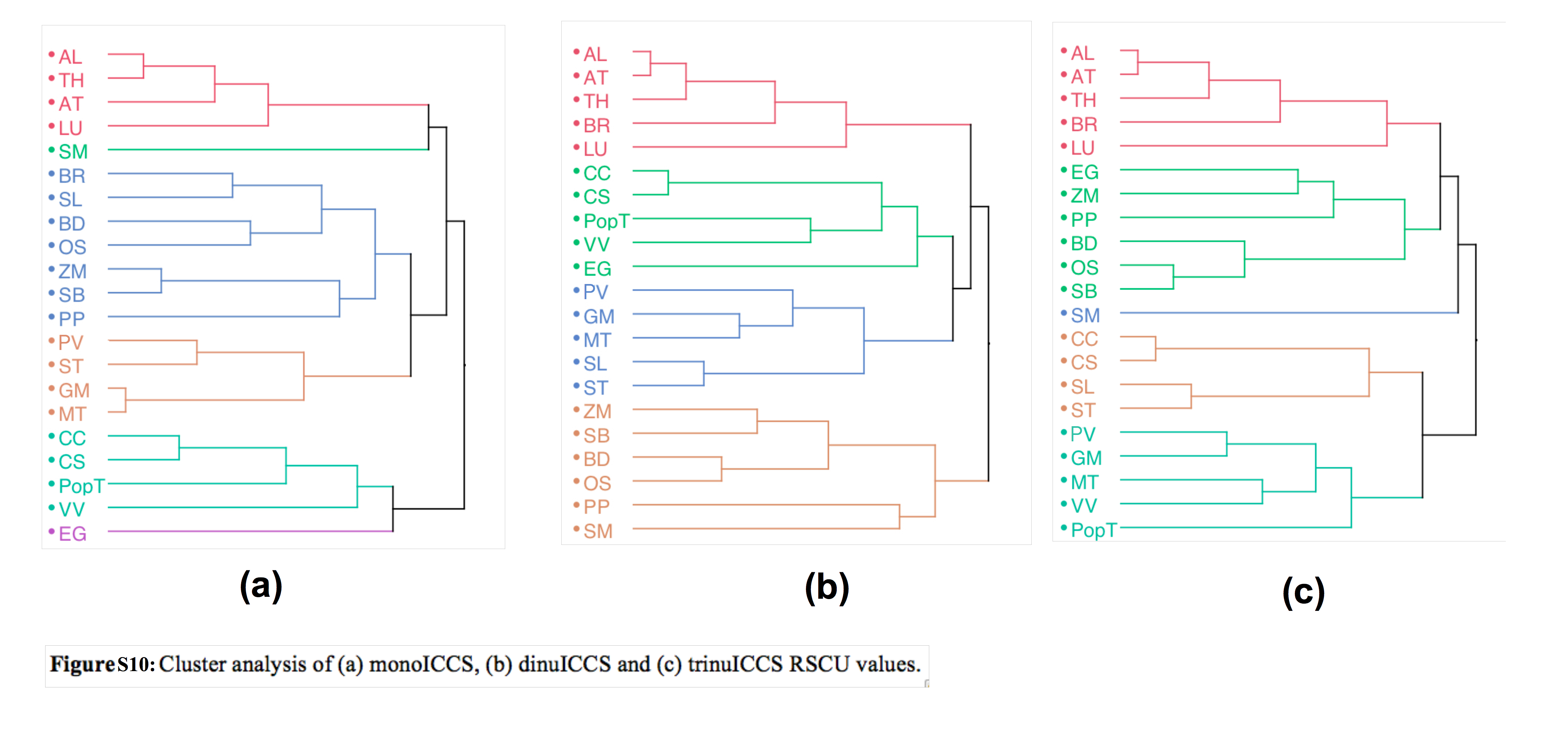

Supplement: Supplementary Data [file supp_dsv027_dsv027supp_fig10.tif]

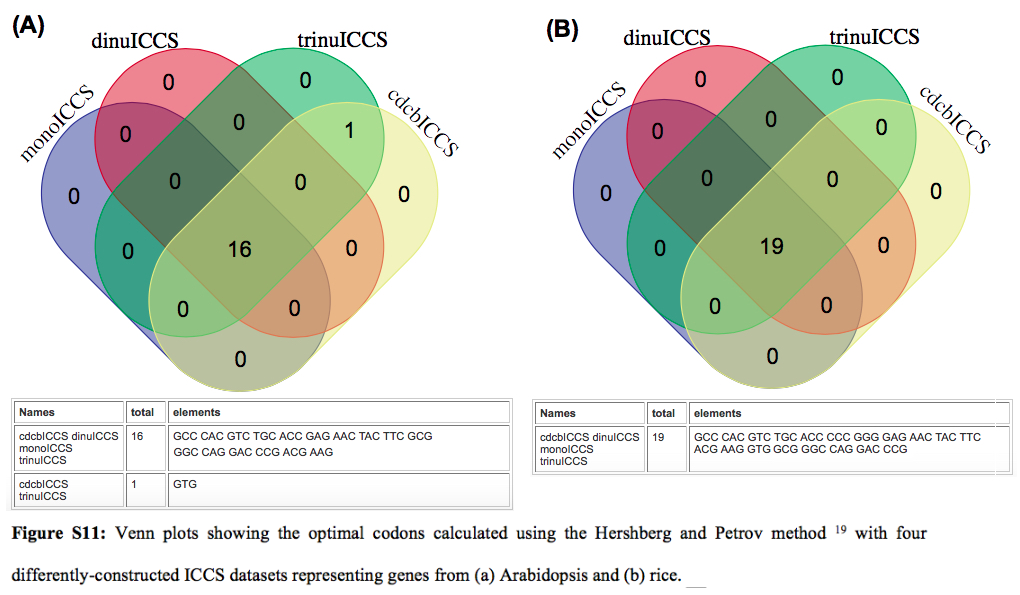

Supplement: Supplementary Data [file supp_dsv027_dsv027supp_fig11.tif]
