# Supplementary material for: New insights into the interplay between codon bias determinants in plants
Source: DNA Res. 2015 Nov 5;22(6):461–70. doi: 10.1093/dnares/dsv027 (PMC4675714; doi:10.1093/dnares/dsv027)
Supplement: Supplementary Data [file supp_dsv027_dsv027supp_table1.pdf]

Amino acid	Frequency	r_{exp}
A	6.16%	0.2877***
C	2.02%	-0.0682***
D	5.14%	-0.0455***
E	6.38%	-0.0845***
F	4.33%	-0.0322***
G	6.32%	0.1705***
H	2.23%	-0.1005***
I	5.27%	-0.0143*
K	6.42%	0.0477***
L	9.12%	-0.1023***
M	2.59%	-0.0208*
N	4.27%	-0.1409***
P	4.80%	0.0469***
Q	3.31%	-0.036***
R	5.39%	-0.0877***
S	9.26%	-0.1556***
T	5.07%	0.0531***
V	6.60%	0.1116***
W	1.23%	-0.0638***
Y	2.81%	0.0151*

Table S1: Relative abundance of amino acids in *A. thaliana* proteins as predicted from coding sequences and correlation with gene expression levels.
